# Traffic-related air pollution exposures and changes in heart rate variability in Mexico City: A panel study

**DOI:** 10.1186/1476-069X-12-7

**Published:** 2013-01-18

**Authors:** Kyra Naumoff Shields, Jennifer M Cavallari, Megan J Olson Hunt, Mariana Lazo, Mario Molina, Luisa Molina, Fernando Holguin

**Affiliations:** 1 Department of Environmental and Occupational Health, University of Pittsburgh, Bridgeside Point I, 100 Technology Drive, Suite 350, Pittsburgh, PA, 15219, USA; 2 Division of Occupational and Environmental Medicine, University of Connecticut Health Center, 270 Farmington Ave., The Exchange, Suite 262, Farmington, Ct. 06032-6210, USA; 3 Department of Biostatistics, University of Pittsburgh, 130 DeSoto Street, Pittsburgh, PA, 15261, USA; 4 Department of Epidemiology, Johns Hopkins Bloomberg School of Public Health, 615 North Wolfe Street, W6508, Baltimore, Maryland, 21205, USA; 5 Department of Chemistry and Biochemistry, University of San Diego, Science & Technology 374, 5998 Alcala Park, San Diego, CA, 92110, USA; 6 Department of Earth, Atmospheric and Planetary Sciences Cambridge, Massachusetts Institute of Technology, MA 02139, 9500 Gilman Dr., MCO332, La Jolla, CA, 92093-0332, USA; 7 Montefiore Hospital, University of Pittsburgh Medical Center, 3459 Fifth Avenue, Pittsburgh, PA, 15213, USA

**Keywords:** Air pollution, PM_2.5_, Ozone, Heart rate variability, Mexico City

## Abstract

**Background:**

While air pollution exposures have been linked to cardiovascular outcomes, the contribution from acute gas and particle traffic-related pollutants remains unclear. Using a panel study design with repeated measures, we examined associations between personal exposures to traffic-related air pollutants in Mexico City and changes in heart rate variability (HRV) in a population of researchers aged 22 to 56 years.

**Methods:**

Participants were monitored for approximately 9.5 hours for eight days while operating a mobile laboratory van designed to characterize traffic pollutants while driving in traffic and “chasing” diesel buses. We examined the association between HRV parameters (standard deviation of normal-to-normal intervals (SDNN), power in high frequency (HF) and low frequency (LF), and the LF/HF ratio) and the 5-minute maximum (or average in the case of PM_2.5_) and 30-, 60-, and 90-minute moving averages of air pollutants (PM_2.5_, O_3_, CO, CO_2_, NO_2_, NO_x_, and formaldehyde) using single- and two-pollutant linear mixed-effects models.

**Results:**

Short-term exposure to traffic-related emissions was associated with statistically significant acute changes in HRV. Gaseous pollutants – particularly ozone – were associated with reductions in time and frequency domain components (*α* = 0.05), while significant positive associations were observed between PM_2.5_ and SDNN, HF, and LF. For ozone and formaldehyde, negative associations typically increased in magnitude and significance with increasing averaging periods. The associations for CO, CO_2_, NO_2_, and NO_x_ were similar with statistically significant associations observed for SDNN, but not HF or LF. In contrast, PM_2.5_ increased these HRV parameters.

**Conclusions:**

Results revealed an association between traffic-related PM exposures and acute changes in HRV in a middle-aged population when PM exposures were relatively low (14 *μ*g/m^3^) and demonstrate heterogeneity in the effects of different pollutants, with declines in HRV – especially HF – with ozone and formaldehyde exposures, and increases in HRV with PM_2.5_ exposure. Given that exposure to traffic-related emissions is associated with increased risk of cardiovascular morbidity and mortality, understanding the mechanisms by which traffic-related emissions can cause cardiovascular disease has significant public health relevance.

## Background

Many studies have demonstrated the association between air pollution exposure, specifically fine particulate matter (PM_2.5_), and increased cardiovascular morbidity and mortality [1-9]. Short-term PM exposures have been linked to acute cardiovascular events including increased odds of having a myocardial infarction, cardiac arrhythmia, and venous thrombosis [10], while long-term exposure to PM has been associated with increased risk and progression of atherosclerosis [11].

While the exact biological mechanism linking exposure to PM and cardiovascular outcomes remains unknown [3,12], alterations in heart rate variability (HRV) are thought to be one of the pathophysiologic pathways whereby PM affects the cardiovascular system [13]. HRV is an indicator of the relative balance of parasympathetic and sympathetic autonomic control of the heart rate, and changes (both increases and decreases) in this metric have been associated with both ambient and traffic-related PM air pollution [14-17].

Typically, higher PM concentrations have been associated with decreased HRV in elderly populations and in patients with current or underlying cardiovascular disease [12,18,19]. Findings from studies on the association between HRV and PM in younger populations, however, have been inconsistent. In a panel study of 76 young college students, HRV indices declined in single-pollutant models with PM_10_, PM_2.5_, sulfate, nitrate, and ozone (O_3_) [20]. In a controlled exposure study (mean age = 27 years), exposure to concentrated ambient particles had no consistent effect on HRV indices [21]. Similarly, no consistent effect of diesel exhaust on HRV was observed in a separate double-blind, crossover, controlled-exposure study (mean age = 32 years for healthy subjects, mean age = 41 years for those with metabolic syndrome) [22]. In this case, the controlled-exposure studies may not have accurately simulated actual environmental conditions. In an occupational panel of young boilermakers (mean age = 38 years), significant increases in an HRV index (standard deviation of normal-to-normal intervals or SDNN) were observed for every 1 *μ*g/m^3^ increase in lead and vanadium concentrations [23]. Cardiovascular comorbidity, such as hypertension, which are more prevalent in older populations, has been shown to increase susceptibility to fine particulate matter-mediated reductions in HRV [24]. This phenomenon may determine why older individuals have a different response to air pollution-mediated changes in cardiac autonomic regulation, when compared to younger persons.

Several studies have demonstrated a stronger association between cardiovascular endpoints and particles originating from traffic as compared to other sources [16,18,25-27]. The study of traffic-related air pollutants, however, is complicated due to the nature of traffic exposures, which may vary over short distances and thus limit the use of centralized exposure monitoring data in epidemiological studies.

To reduce the potential for exposure misclassification of traffic-related emissions, several studies have monitored in-vehicle pollutant exposure in young populations. Increases in HRV were observed in association with in-vehicle PM_2.5_ exposure in a group of young North Carolina State Highway Patrol troopers (mean age = 27 years) [28]. In a population of young, highly-exposed taxi drivers in Beijing (mean age = 36 years), low PM_2.5_ exposures were associated with relatively high HRV, whereas higher PM_2.5_ exposures were associated with relatively low HRV [12].

In addition to the discrepancy in the relationship between PM exposure and HRV response, there is a lack of knowledge about the health effects due to the potential synergy between PM_2.5_ and ambient gaseous co-pollutants [29]. Several studies have simultaneously evaluated the association of traffic-related PM and gaseous pollutants with HRV [12,21,22,28,30,31]. This is particularly important as exposure to traffic-related emissions in close proximity is characterized by a rich mixture of fine PM and gaseous pollutants that are different from the mixture of the background air pollution exposure [32].

To address the aforementioned gaps in the literature, we examined the association between real-time, traffic-related air pollution exposures and acute sub-clinical cardiovascular outcomes in a middle-aged population. We used a panel study design with repeated measures to account for personal factors while enhancing the statistical power to detect associations through high and variable exposure levels. Our hypothesis was that there is an inverse exposure-response relationship between HRV parameters and traffic-related pollutants including PM_2.5_, carbon dioxide (CO_2_), carbon monoxide (CO), nitrogen dioxide (NO_2_), nitrogen oxides (NO_x_), O_3_, and formaldehyde.

## Methods

### Participants and study design

This repeated-measures panel study was conducted February 11–23, 2002, in the Mexico City Metropolitan Area as part of an effort is to contribute to the understanding of the air quality problem in megacities by conducting measurements and modeling studies of atmospheric pollutants [33]. A convenience sample of the sixteen researchers, between the ages of 22 and 56 years, associated with the project participated in the study. A self-administered questionnaire was used to collect personal data, including sex, age, smoking status and hypertension history. The study design and methods were reviewed and approved by the human subjects committee at the National Institute of Public Health. All participants signed an informed consent form before participating in the study.

Real-time measurements of PM_2.5_, CO_2_, CO, NO_2_, NO_x_, O_3_, and formaldehyde were collected in a van-based mobile laboratory [34]. The van pursued specific vehicles to measure their emissions, drove transects across the city to capture the spatial variation in pollutants, and parked at several locations in the city [35]. The nine individual mobile episodes lasted 1–10 hours. The van drove past a variety of point sources throughout the city, including residential (e.g. biomass burning), industrial (e.g. metal welding, factories, oil burning), livestock, landfill, and sewage treatment sources.

During data collection, approximately six participants at a time wore an Aria Digital Holter Monitor and rode in the van or stood outside the van when it was in a stationary location. In-vehicle and ambient air exchange (and corresponding participant exposures) occurred through frequently open windows in the van’s cab and ambient air penetration through air conditioning vents and through open doors during the van’s frequent short stops during day and longer stops for meals.

### Air pollutant measurements

All air pollution measurements were taken in a van-based mobile laboratory developed by Aerodyne Research Inc. [34,36]. The van was designed to sample and characterize mobile and fixed-site emission plumes, as well as characterize gaseous and particulate emissions from selected classes of vehicles, including heavy-duty diesel trucks, buses, and colectivos (ubiquitous small gasoline or condensed natural gas powered microbuses). It was outfitted with state-of-the-art, fast-response instruments, including a non-dispersive infrared (NDIR) unit (Li-Cor LI 6262) for CO_2_, an Aerodyne tunable infrared laser differential absorption spectrometer (TILDAS) for NO_2_ and formaldehyde (HCHO), a NDIR analyzer for CO, a chemiluminscent analyzer (Thermo 42C) for nitrogen oxides (NO_x_), a UV monitor (Thermo Environmental 49–003) for O_3_[37], and an aerosol photometer for PM_2.5_ (TSI Dustrak 8520) [38].

Sampler inlets were positioned well above and forward of the vehicle engine and generator exhaust outlets [34]. The Dustrak was mounted on a shelf in the van, and the stainless steel and Tygon sampling lines were designed to minimize particle deposition [35].

The intrinsic measurement period varied depending on the type of instrument used. The basic measurement interval was ~1 second for CO_2_, NO_2_, NO_x_, formaldehyde and PM_2.5_; the interval increased to ~20 seconds for O_3_ and CO. Except for a few power or computer loss periods, the instruments measured and recorded continuously. For each gaseous pollutants, i.e. O_3_, CO, CO_2_, NO_2_, NO_x_, and formaldehyde, the maximum value in a given 5-minute window was recorded. From these 5-minute maxes, 30-, 60-, and 90-minute average maximums were calculated (by averaging the 5-minute maxes). For PM_2.5_, 5-minute mean concentrations were recorded and 30-, 60-, and 90-minute means were calculated from these values.

The PM_2.5_ measurements reflected some uncertainty resulting from the calibration of the aerosol photometer [38]. The Dustrak was calibrated against multiple 24-hour PM_2.5_ gravimetric samples throughout the field campaign and applied to the factory-calibrated readings (field calibration factor was 0.34±0.02). This method depends on scattering efficiencies, which, in turn, are a function of optical properties and particle size distributions. Though scattering efficiencies of ambient and diesel particles are similar, gasoline particles may differ in this efficiency, which may cause the calibration for individual vehicles to vary by a factor of two or more.

Additionally, distinct “spike events” in the UV O_3_ monitor on board the van were observed when the van was sampling the ambient diluted exhaust from on-road diesel vehicles [37]. Associated fine particles were assumed to have caused the observed interference. This type of interference could lead to a mean measured O_3_ concentration that is at most 3% higher than actual concentrations.

### Heart rate variability measurements

HRV was obtained from analysis of the ambulatory electrocardiogram recorded using an Aria Digital Holter Monitor (Del Mar Reynolds, US). Participants were allowed to participate on multiple occasions up to eight days for a total of 48 person-days. Electrodes were placed on the right parasternal and precordial areas, and continuous monitoring occurred between 7:05 and 20:05 with the average monitoring period occurring from 9:30 to 19:00. HRV parameters were calculated in 5-minute epochs and abnormal or ectopic beats were manually removed. Digitized Aria Digital Halter recordings were analyzed using a Marquette MARS Workstation that provided an algorithm for HRV analysis and interpolation for removed aberrant QRS complexes (Del Mar Reynolds, US).

HRV parameters included the standard deviation of normal R-R internals (SDNN), which is a time domain measure of overall HRV, and high- (0.18-0.40 Hz) and low-frequency power (0.03-0.15 Hz), which are respectively representative of predominantly parasympathetic and sympathetic autonomic cardiac regulation. (The LF/HF ratio represents the relative balance between sympathetic-vagal nervous activity [39].

### Statistical analysis

All heart rate variability measures were log-transformed using the natural logarithm to help meet regression assumptions. No predictors were transformed, as model assumptions were reasonably met by transforming the outcome variables only.

The associations between each of the HRV parameters and individual pollutants were examined using linear mixed-effects models (*α* = 0.05). The gaseous pollutants O_3_, CO, and NO_2_ were also individually evaluated in two-pollutant models with PM_2.5_, and potential multicollinearity between the two pollutants was assessed using the condition number [40]. A condition number greater than 30 suggests moderate multicollinearity could be present, whereas a value over 100 indicates severe multicollinearity is likely occurring. Multicollinarity is a concern because it may affect the stability of point estimates and the accuracy of their inference, leading to incorrect conclusions about associations between a set of predictors and an outcome.

A random subject intercept was incorporated into each model with an exponential covariance structure to account for the unevenly spaced 5-minute measurements over 12 hours, performed over eight unequally spaced days. The exponential covariance structure allows for the correlation within the same subject to decay over time [41]. All analyses were conducted using one or two air pollutant models over periods of 5, 30, 60, and 90 minutes, as described previously.

Models were adjusted for fixed and time-varying factors that were potential confounders. Fixed factors included sex, age (linear), smoking status, and ethnic origin (Mexican or other). Time-varying factors included a categorical variable for time of day (06:00 to 11:59, 12:00 to 15:59, and 16:00 to 20:05), and study day (eight categories). To examine the influence of outlying exposure values, the smallest and largest 5% of pollutant values for a given averaging period (10% total) were excluded in a separate analysis [12].

Final results (*β*-values) are presented as the estimated percent change of a given HRV outcome per interquartile range (IQR) increase in the exposure to each air pollutant, controlling for sex, age, smoking status, ethnic origin, time of day, and study day. Estimates were calculated as *β* = [exp(*β′* × IQR) – 1] × 100%, where *β′* was the estimated effect of a pollutant from the mixed-model [12]. Similarly, the 95% confidence interval was achieved from the following transformation: [exp(IQR × CI*′*)– 1] × 100%, where CI*′* represents the estimated 95% confidence interval for *β′* from the mixed-model. Statistical analyses were performed in R (version 2.13.2) and SAS (version 9.2).

## Results

Table 1 includes detailed information on the characteristics of the 16 study participants, including SDNN, high- and low-frequency spectral HRV domain (HF and LF, respectively), and the ratio of LF/HF. The majority (69%) were males. Fifty percent of participants were of Mexican ethnic origin; the remaining were Caucasian American. Participants’ ages ranged from 22 to 56 years, with a mean of 35 years (SD = 11.8) and a median of 31 (IQR = 16.5). The majority were non-smokers (87%), with only one individual reporting hypertension (6%).

**Table 1:** Basic characteristics and the outcome measures of the sixteen study participants, Mexico City Metropolitan Area, February 2002

***Demographics***		***n *(%)**	
Male		11 (68.8)	
Mexican		8 (50)	
Smoker		2 (12.5)	
Former smoker		4 (25)	
Hypertension		1 (6.3)	
Age (years)			
Mean (SD)		34.6 (11.8)	
Median (IQR)		30.5 (16.5)	
Range		22-56	
***Heart Rate Variability Measures***			
***n *= 4436 for all variables (total number of measures across all subjects)**			
	**Mean (SD)**	**Median (IQR)**	**Range**
SDNN (msec)	66.6 (28.9)	62.0 (29.9)	4.1-704.9
HF (Hz)	234.4 (458.5)	121.9 (213.5)	0.3-8535.0
LF (Hz)	731.0 (591.4)	592.5 (639.3)	1.0-7155.0
LF/HF	6.7 (6.0)	4.9 (5.8)	0.1-130.6

Table 2 shows the exposure characteristics over different periods (5-, 30-, 60-, and 90-min intervals) for PM_2.5_, O_3_, CO, CO_2_, formaldehyde, NO_2_, and NO_x_. NO_2_ (mean: 130 ppb) exceeded the one-hour National Ambient Air Quality Standard (NAAQS) concentration (100 ppb) [42]. CO mean one-hour exposure (6 ppm) was below the corresponding NAAQS (35 ppm).

**Table 2:** Average measured pollutant concentrations over different time periods in the Mexico City Metropolitan Area, February 2002

	**5-minute**		**30-minute**		**60-minute**		**90-minute**	
	**Mean**	**Median**	**Mean**	**Median**	**Mean**	**Median**	**Mean**	**Median**
	**( *n *, SD)**	**(IQR)**	**( *n *, SD)**	**(IQR)**	**( *n *, SD)**	**(IQR)**	**( *n *, SD)**	**(IQR)**
PM_2.5_, mean (*μ*g/m^3^)	14	11	14	12	14	12	14	12
	(3314, 12)	(10)	(3140, 9)	(11)	(2939, 8)	(9)	(2733, 8)	(8)
Ozone, max (ppb)	84	69	85	73	87	77	89	83
	(4406, 66)	(65)	(4118, 52)	(66)	(3832, 46)	(73)	(3550, 43)	(65)
CO, max (ppm)	6	2	6	2	6	3	6	3
	(4353, 8)	(8)	(4086, 7)	(10)	(3806, 6)	(10)	(3524, 6)	(9)
CO_2_, max (ppm)	461	426	462	431	462	433	462	432
	(4371, 97)	(96)	(4088, 79)	(106)	(3802, 73)	(104)	(3520, 69)	(105)
Formaldehyde, max (ppb)	35	23	34	25	35	26	35	27
	(1784, 39)	(26)	(1663, 26)	(30)	(1519, 24)	(31)	(1379, 23)	(34)
NO_2_, max (ppb)	130	68	131	69	130	71	128	74
	(4406, 135)	(114)	(4118, 121)	(137)	(3832, 114)	(155)	(3550, 108)	(156)
NO_x_, max (ppb)	131	23	131	23	130	25	128	25
	(4406, 187)	(186)	(4118, 176)	(240)	(3832, 169)	(237)	(3550, 161)	(226)

Sample sizes are the total number of repeated observations across all 16 subjects.

Table 3 gives Spearman’s rank correlation coefficients for the exposure variables from the 5-min time period. Several of the pollutant measurements were strongly correlated (i.e. *ρ* > 0.70): CO_2_ and CO, formaldehyde and CO, formaldehyde and CO_2_, NO_2_ and CO, NO_2_ and CO_2_, NO_x_ and CO, NO_x_ and CO_2_, and NO_x_ and NO_2_.

**Table 3:** Spearman’s rank correlation coefficients for exposure variables from the 5-minute time period

	**PM**_**2.5**_	**Ozone**	**CO**	**CO**_**2**_	**Formaldehyde**	**NO**_**2**_	**NO**_**x**_
PM_2.5_ (*n* = 3314)	1.0						
Ozone (*n* = 4406)	0.33	1.0					
CO (*n* = 4353)	0.24	−0.07	1.0				
CO_2_ (*n* = 4371)	0.27	−0.06	0.78	1.0			
Formaldehyde (*n* = 1784)	0.56	0.36	0.79	0.77	1.0		
NO_2_ (*n* = 4406)	0.21	−0.007	0.84	0.72	0.60	1.0	
NO_x_ (*n* = 4406)	−0.13	−0.34	0.79	0.70	0.52	0.81	1.0

Sample sizes are the total number of repeated observations across all 16 subjects.

**Figure 1:**
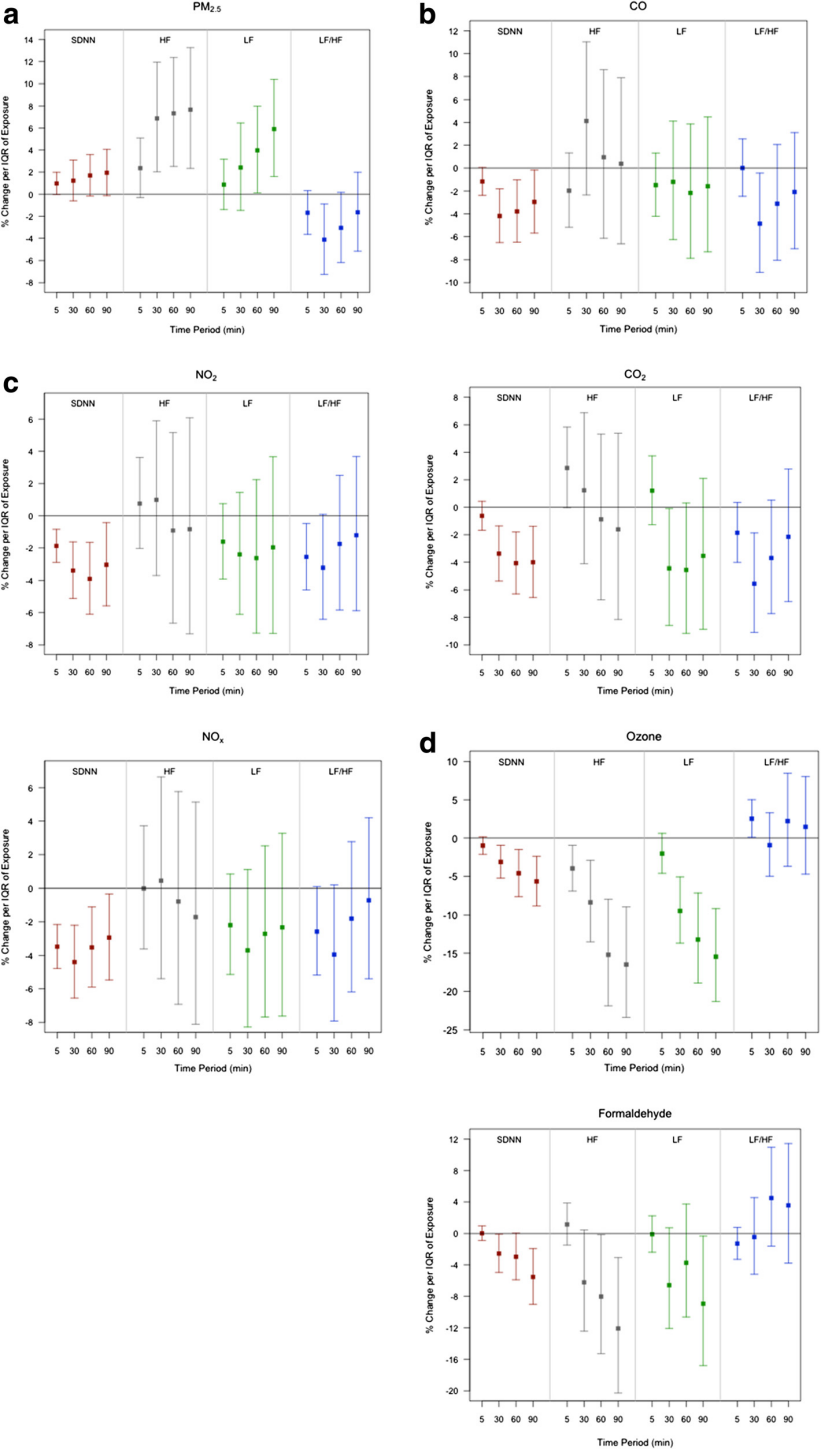
**a. Point estimates and 95% confidence intervals for PM**_**2.5**_**.** Positive associations were observed for PM_2.5_ and SDNN, HF and LF. A negative association was observed for the LF/HF ratio. The largest increase observed wasa 7.7% (95% CI: 2.3 to 13.3) increase in HF per IQR 90-min PM_2.5_ (8.34 *μ*g/m^3^). Figure 1**b**. Point estimates and 95% confidence intervals for CO and CO2. The largest declines in SDNN were 4.2% (95% CI: 1.8 to 6.5) per IQR 30-min CO (10 ppm) and 4.1% (95% CI: 1.8 to 6.3) per IQR 60-min CO2 (104 ppm). Figure 1**c**. Point estimates and 95% confidence intervals for NO¬2 and NOx. The associations for CO, CO2, NO2 and NOx (which were all correlated) were similar with statistically significant associations observed for SDNN and, in a few instances, the LF/HF ratio, but not HF or LF. The influence of averaging period differed for each pollutant. The largest declines in SDNN for each pollutant were a 3.9% (95% CI: 1.7 to 6.1) per IQR 60-min NO2 (155ppb), and 4.4% (95% CI: 2.2 to 6.5) per IQR 30-min NOx (240 ppb). Figure 1**d**. Point estimates and 95% confidence intervals for ozone and formaldehyde. Associations increased in magnitude and significance with increasing averaging periods for all HRV parameters except the LF/HF ratio. The largest effects were observed for HF, including a 16% (95% CI: 9.0 to 23.4) decline per IQR 90-min ozone (65 ppb) and a 12% (95% CI: 3.1 to 20.3) decline per IQR 90-min formaldehyde (34 ppb).

We estimated associations between the HRV measures and exposure to pollutants over different moving averages (from 5 to 90 min) after adjusting for potential confounders (Table 4 and Figure 1). Positive associations were observed between HRV parameters (SDNN, HF, and LF) and PM_2.5_ exposures, but the LF/HF ratio was negatively associated with PM_2.5_. The largest percent increases were observed for HF over the 90-min averaging period: a 7.74% (95% CI: 2.3 to 13.3) increase in HF was found per IQR 90-min PM_2.5_ (8.3 *μ*g/m^3^).

**Table 4:** **Results from single**-**pollutant linear mixed**-**models: *β*-values are percent change in the outcome per IQR increase in exposure, controlling for sex, age, smoking status, ethnic origin, time of day, and study day**

			**SDNN**		**HF**		**LF**		**LF/HF**	
	***n***	**IQR**	***β***	***p*-value**	***β***	***p*-value**	***β***	***p*-value**	***β***	***p*-value**
PM_2.5_ (*μ*g/m^3^)										
5-min	3314	9.82	0.97	0.06	2.36	0.08	0.86	0.46	−1.69	0.10
30-min	3140	10.5	1.22	0.19	6.87	0.005^†^	2.42	0.23	−4.12	0.01^†^
60-min	2939	8.72	1.70	0.08	7.33	0.003^†^	3.97	0.04^†^	−3.05	0.06
90-min	2733	8.34	1.94	0.07	7.66	0.004^†^	5.90	0.007^†^	−1.64	0.37
Ozone (ppm)										
5-min	4406	65.2	−0.98	0.09	−3.96	0.01^†^	−2.03	0.13	2.53	0.04^†^
30-min	4118	66.4	−3.12	0.005^†^	−8.36	0.003^†^	−9.48	<0.0001^†^	−0.93	0.66
60-min	3832	73.5	−4.60	0.004^†^	−15.20	0.0001^†^	−13.22	<0.0001^†^	2.22	0.47
90-min	3550	65.0	−5.66	0.0008^†^	−16.48	<0.0001^†^	−15.45	<0.0001^†^	1.47	0.65
CO (ppm)										
5-min	4353	7.60	−1.17	0.06	−1.98	0.24	−1.49	0.30	0.01	0.99
30-min	4086	10.2	−4.19	0.0006^†^	4.13	0.22	−1.20	0.65	−4.86	0.03^†^
60-min	3806	10.5	−3.79	0.008^†^	0.95	0.80	−2.17	0.47	−3.12	0.23
90-min	3524	9.35	−2.96	0.04^†^	0.38	0.92	−1.59	0.60	−2.09	0.42
CO_2_ (ppm)										
5-min	4371	96.3	−0.63	0.24	2.86	0.053	1.21	0.34	−1.86	0.10
30-min	4088	106	−3.38	0.001^†^	1.23	0.66	−4.44	0.045^†^	−5.55	0.003^†^
60-min	3802	104	−4.07	0.0005^†^	−0.88	0.78	−4.55	0.07	−3.69	0.08
90-min	3520	105	−4.00	0.003^†^	−1.61	0.64	−3.54	0.21	−2.15	0.39
Formaldehyde (ppb)										
5-min	1784	25.5	0.03	0.94	1.16	0.39	−0.09	0.94	−1.27	0.22
30-min	1663	29.8	−2.55	0.04^†^	−6.21	0.07	−6.58	0.03^†^	−0.44	0.86
60-min	1519	31.0	−2.95	0.06	−8.03	0.047^†^	−3.72	0.32	4.49	0.15
90-min	1379	34.4	−5.53	0.003^†^	−12.09	0.01^†^	−8.94	0.04^†^	3.55	0.35
NO_2_ (ppb)										
5-min	4406	114	−1.87	0.0004^†^	0.75	0.60	−1.61	0.18	−2.56	0.02^†^
30-min	4118	137	−3.39	0.0002^†^	0.99	0.69	−2.40	0.22	−3.22	0.06
60-min	3832	155	−3.90	0.0007^†^	−0.92	0.76	−2.63	0.28	−1.75	0.41
90-min	3550	156	−3.04	0.02^†^	−0.84	0.81	−1.96	0.49	−1.21	0.62
NO_x_ (ppm)										
5-min	4406	186	−3.48	<0.0001^†^	−0.01	0.995	−2.20	0.15	−2.58	0.06
30-min	4118	240	−4.40	0.0001^†^	0.44	0.88	−3.70	0.13	−3.95	0.06
60-min	3832	237	−3.53	0.005^†^	−0.78	0.81	−2.71	0.30	−1.81	0.43
90-min	3550	226	−2.94	0.03^†^	−1.71	0.62	−2.33	0.41	−0.71	0.77

Sample sizes are the total number of repeated observations across all 16 subjects.

No positive associations were found between O_3_, CO, CO_2_, NO_2_, NO_x_, and formaldehyde exposures and SDNN, HF, and LF. For ozone and formaldehyde, negative associations increased in magnitude and significance with increasing averaging periods for SDNN, HF, and LF (ozone only). The largest effect for each pollutant was observed for HF, with a 16% (95% CI: 9.04 to 23.4) decline per IQR 90-min ozone (65 ppb) and a 12% (95% CI: 3.1 to 20.3) decline per IQR 90-min formaldehyde (34 ppb). The associations for CO, CO_2_, NO_2,_ and NO_x_ (which were all correlated) were similar with statistically significant associations observed for SDNN. The influence of averaging period differed for each pollutant, and we observed the largest declines in SDNN for each pollutant as follows: 4.2% (95% CI: 1.8 to 6.5) per IQR 30-min CO (10 ppm), 4.1% (95% CI: 1.8 to 6.3) per IQR 60-min CO_2_ (104 ppm), 3.9% (95% CI: 1.7 to 6.1) per IQR 60-min NO_2_ (155 ppb), and 4.4% (95% CI: 2.2 to 6.5) per IQR 30-min NO_x_ (240 ppb). For this same group of four pollutants, no statistically significant associated were observed for HF, LF, or the LF/HF ratio, with the exception of significance between CO_2_ and HF and LF.

Across all HRV outcomes and pollutants, study day and time of day were significant predictors of the outcome. Ethnic origin and age were sometimes significant, but gender and smoking status were never significant predictors.

We investigated potential confounding by the gaseous pollutants O_3_, CO, and NO_2_ by including each of them individually in a two-pollutant model with PM_2.5_ (Table 5). The largest condition number (a measure of multicollinearity) for the fixed effects in the two-pollutant models was 18.05 (for PM_2.5_ and CO with a 30-min averaging period), and the values across all models were similar in magnitude. Condition numbers of this size indicate multicollinearity could be having a weak effect on the coefficient estimates [40]. However, since a value greater than 100 is often used as a benchmark for significant multicollinearity, estimation and inference likely wasn’t affected in this instance.

**Table 5:** **Results from two-pollutant linear mixed-models: *β*-values are percent change in the outcome per IQR increase in a given pollutant, controlling for sex, age, smoking status, ethnic origin, time of day, study day, and the other pollutant in the model**

			**SDNN**		**HF**		**LF**		**LF/HF**	
	***n***	**IQR**	***β***	***p*-value**	***β***	***p*-value**	***β***	***p*-value**	***β***	***p*-value**
PM_2.5_ (*μ*g/m^3^) Ozone (ppm)										
5-min	3292	9.82	0.96	0.06	2.41	0.08	0.84	0.47	−1.77	0.08
		65.2	−0.55	0.50	−3.82	0.07	−2.33	0.20	1.88	0.25
30-min	3118	10.5	1.21	0.20	6.82	0.005^†^	2.41	0.22	−4.09	0.01^†^
		66.4	−4.51	0.001^†^	−7.81	0.02^†^	−8.65	0.003^†^	−0.81	0.75
60-min	2917	8.72	1.80	0.06	7.66	0.002^†^	4.15	0.03^†^	−3.16	0.06
		73.5	−6.97	0.0003^†^	−13.59	0.003^†^	−11.63	0.002^†^	1.98	0.58
90-min	2711	8.34	2.29	0.03^†^	8.13	0.003^†^	6.53	0.003^†^	−1.49	0.42
		65.0	−6.35	0.002^†^	−14.64	0.002^†^	−15.66	0.0001^†^	−1.03	0.78
PM_2.5_ (*μ*g/m^3^) CO (ppm)										
5-min	3240	9.82	1.02	0.049^†^	2.37	0.08	0.83	0.47	−1.73	0.09
		7.60	−1.32	0.09	−1.79	0.37	−2.45	0.15	−1.16	0.45
30-min	3086	10.5	1.17	0.22	6.28	0.01^†^	2.14	0.28	−3.84	0.02^†^
		10.2	−2.66	0.08	9.16	0.02^†^	−1.52	0.63	−9.51	0.0003^†^
60-min	2891	8.72	1.45	0.14	6.49	0.009^†^	3.55	0.08	−2.69	0.11
		10.5	−1.77	0.32	5.81	0.20	−2.38	0.50	−7.75	0.01^†^
90-min	2685	8.34	1.83	0.09	6.94	0.01^†^	5.65	0.01^†^	−1.17	0.53
		9.35	−0.59	0.74	4.87	0.27	−1.34	0.70	−6.19	0.04^†^
PM_2.5_ (*μ*g/m^3^) NO_2_ (ppb)										
5-min	3292	9.82	1.08	0.04^†^	2.30	0.09	0.97	0.40	−1.53	0.13
		114	−2.06	0.004^†^	0.54	0.77	−2.68	0.09	−3.41	0.01^†^
30-min	3118	10.5	1.36	0.15	6.61	0.007^†^	2.56	0.20	−3.76	0.02^†^
		137	−2.29	0.05^†^	4.02	0.19	−1.82	0.46	−5.52	0.007^†^
60-min	2917	8.72	1.75	0.07	7.32	0.003^†^	4.01	0.04^†^	−2.98	0.07
		155	−2.07	0.16	4.54	0.23	−1.34	0.65	−5.59	0.03^†^
90-min	2711	8.34	1.98	0.06	7.38	0.006^†^	5.71	0.009^†^	−1.54	0.40
		156	−1.06	0.53	4.71	0.26	−0.19	0.96	−4.89	0.09

Each cell contains the value for each of the two pollutants in a given model (in the order given in the left most column). Sample sizes are those used in the regression models and are the total number of repeated observations across all 16 subjects.

In general, adjusting for O_3_ had little effect on the PM_2.5_ estimates across all HRV outcomes. The magnitudes of the PM_2.5_ estimates tended to fluctuate only slightly and the directions never changed. The significance of the results did not change with the exception of the 90-min window and SDNN (which became significant in the two-pollutant model).

The findings after adjusting for CO were similar. The PM_2.5_ magnitudes did not change notably and the directions of the estimates remained consistent. For SDNN 5-min and LF 60-min, the significance of the PM_2.5_ estimates in the two-pollutant models differed from those in the single-pollutant, while all others remained unchanged.

Lastly, adjusting for NO_2_, the PM_2.5_ estimate associated with the 5-min window and SDNN was not significant in the single-pollutant model, but significant in the two-pollutant model. No other results changed. As with the other gaseous confounders, adjusting for NO_2_ had little or no effect on the size or direction of the PM_2.5_ estimates.

To evaluate the influence of outlying exposure values on reported associations, we removed observations containing the highest and lowest 5% of pollutant concentrations (10% total). An average of 322 data points were removed for a given model, while an average of 2988 remained (across all individuals) (Table 6). Within a given individual, the smallest number of observations was 20 for this analysis.

**Table 6:** Results from single-pollutant linear mixed-model sensitivity analysis: Observations with the smallest and largest 5% of pollutant values (10% total) were removed to evaluate the influence of outlying exposure values on the observed associations

			**SDNN**		**HF**		**LF**		**LF/HF**	
	***n***	**IQR**	***β***	***p*-value**	***β***	***p*-value**	***β***	***p*-value**	***β***	***p*-value**
PM_2.5_ (*μ*g/m^3^)										
5-min	2982	8.43	2.30	0.01^†^	0.81	0.73	1.99	0.33	1.22	0.50
30-min	2826	8.62	0.09	0.92	1.71	0.48	0.46	0.82	−1.32	0.45
60-min	2645	7.28	0.43	0.61	2.12	0.34	1.76	0.36	−0.48	0.77
90-min	2459	6.56	0.33	0.70	3.58	0.12	2.14	0.26	−1.44	0.38
Ozone (ppm)										
5-min	3964	58.5	−0.40	0.72	−13.17	< 0.0001^†^	−9.67	0.0001^†^	4.18	0.08
30-min	3706	60.2	−2.07	0.06	−10.72	0.0001^†^	−7.82	0.001^†^	3.28	0.15
60-min	3448	62.8	−3.75	0.002^†^	−13.78	< 0.0001^†^	−11.06	< 0.0001^†^	3.16	0.23
90-min	3194	60.0	−6.23	< 0.0001^†^	−19.14	< 0.0001^†^	−16.86	< 0.0001^†^	2.96	0.31
CO (ppm)										
5-min	3917	5.60	−2.15	0.0001^†^	1.56	0.31	−0.82	0.52	−2.37	0.04^†^
30-min	3676	8.57	−3.18	0.0006^†^	3.03	0.22	−1.44	0.49	−4.36	0.02^†^
60-min	3424	8.88	−3.53	0.0007^†^	0.23	0.93	−2.52	0.28	−2.78	0.21
90-min	3170	7.98	−3.52	0.0003^†^	−2.39	0.37	−2.35	0.31	0.07	0.98
CO_2_ (ppm)										
5-min	3933	77.8	−1.81	0.007^†^	1.11	0.54	−2.35	0.13	−3.43	0.01^†^
30-min	3678	90.2	−2.06	0.02^†^	0.34	0.89	−2.55	0.20	−2.89	0.11
60-min	3420	90.9	−3.75	0.0001^†^	−2.27	0.38	−4.72	0.03^†^	−2.29	0.24
90-min	3168	92.3	−4.86	< 0.0001^†^	−4.74	0.11	−7.26	0.004^†^	−2.47	0.27
Formaldehyde (ppb)										
5-min	1604	20.9	−0.81	0.31	1.93	0.39	−1.74	0.38	−1.74	0.03^†^
30-min	1495	26.3	−1.18	0.31	−1.52	0.63	−2.01	0.47	−0.52	0.83
60-min	1367	24.6	−2.12	0.08	−6.29	0.04^†^	−5.22	0.05^†^	1.00	0.69
90-min	1241	28.8	−4.22	0.006^†^	−10.22	0.007^†^	−8.24	0.02^†^	2.34	0.47
NO_2_ (ppb)										
5-min	3964	95.3	−2.85	< 0.0001^†^	1.13	0.41	−2.46	0.03^†^	−3.49	0.0006^†^
30-min	3706	111	−2.49	0.0001^†^	1.17	0.52	−1.95	0.20	−3.05	0.03^†^
60-min	3448	136	−2.40	0.008^†^	0.77	0.75	−1.96	0.34	−2.58	0.15
90-min	3194	136	−2.69	0.006^†^	−0.91	0.73	−1.36	0.56	−0.24	0.90
NO_x_ (ppm)										
5-min	3964	121	−3.07	< 0.0001^†^	−0.83	0.46	−2.29	0.02^†^	−1.48	0.09
30-min	3706	172	−3.37	< 0.0001^†^	−1.44	0.43	−3.03	0.06	−1.61	0.26
60-min	3448	197	−4.02	< 0.0001^†^	−2.10	0.34	−4.03	0.04^†^	−1.85	0.28
90-min	3194	200	−3.61	< 0.0001^†^	−3.83	0.13	−3.34	0.14	0.58	0.78

*β*-values are percent change in the outcome per IQR increase in exposure, controlling for sex, age, smoking status, ethnic origin, time of day, and study day. Sample sizes are the total number of repeated observations across all 16 subjects.

^†^ Significant with *α* = 0.05.

The associations between PM_2.5_ and HF for the 30-, 60-, and 90-min averaging periods were smaller in magnitude and no longer significant after removal of the high/low values, suggesting these associations may be influenced by extreme exposure values. For other pollutants, the reported IQR systematically decreased, but the direction and significance of the estimates generally remained the same.

## Discussion

Short-term exposure to traffic-related emissions was associated with significant acute changes in HRV in this panel study of researchers that participated in the Mexico City Air Pollution Campaign [33]. Gaseous pollutants – particularly ozone – were associated with reductions in time and frequency domain components. In contrast, PM_2.5_ increased these HRV parameters. Like Riediker [28], our results show a positive association between PM_2.5_ (mean PM_2.5_ mass concentration = 23.0 μg/m^3^) and two frequency domain HRV parameters (HF and LF), a result contrary to those observed in multiple studies of elderly populations exposed to PM air pollution related to traffic [13,43,44] (median PM_2.5_ mass concentration in each study, respectively = 10 μg/m^3^, 8.92 μg/m^3^ (2-hr), 7.7 μg/m^3^ (5 minute). Our results also demonstrate a negative association between the LF/HF ratio and PM_2.5_, CO, and CO_2_ that is significant for the 30-min averaging period. When assessed in normalized units (i.e. ratio), LF and HF provide quantitative indicators of neural control of the sinoatrial node and provide a synthetic index of the sympathovagal balance [45], which may be predictive of the development of ventricular arrhythmias [46]. Our results, similar to other studies discussed below, suggest that the air pollution-associated acute changes in HRV parameters (increased or reduced) that have the highest potential for increasing the likelihood of a subsequent cardiac arrhythmia remain to be determined. Nonetheless, our results have significant implications to our understanding of how air pollution leads to an increase risk of cardiac arrhythmias. While having a reduced HRV is a risk factor for increased cardiovascular mortality; it is yet to be shown whether short-term environmental exposures associated with acute HRV changes can lead to cardiac arrhythmias in humans. This pathway, however, seems biologically plausible given that a) acute reductions or increases in HRV parameters have been associated with the onset of ventricular tachycardia [47-49], and b) short-term increases in ambient pollutants (both particles and gases) have been associated with increased likelihood of having a discharge from an implantable cardiac defibrillator [50].

Wu et al. [12] measured real-time, in-vehicle, traffic-related PM_2.5_ (56.6 μg/m^3^ (daily average)), and gaseous co-pollutants (CO, NO_2,_ and NO) in a young population (*n* = 11, mean age = 35.5 years). They showed that IQR increases in PM_2.5_ mass concentrations (5–240 min moving averages) were associated with declines in three, 5-min HRV indices (SDNN, LF, and HF). Results, however, from their regression models for each subject showed heterogeneity among responses, i.e. several subjects in the study had positive associations with traffic-related PM exposures for the three, 5-min HRV indices. Further, their smoothed curves showing associations between PM exposure and 5-min HRV indices indicated that lower PM exposures were associated with increases in HRV, whereas higher PM exposures were associated with decreases in HRV. Similarly, to explore potential confounding by the gaseous co-pollutants, we separately included CO, NO_x_, and NO in a two-pollutant model with PM_2.5_. While adjusting for the co-pollutants decreased the precision of the estimates, our overall results were generally consistent with estimates not adjusted for co-pollutants. Overall, the results of Wu et al., like ours, suggest that differences in HRV response to traffic-related PM pollution may be related to differences in exposure levels, though factors impacting the heterogeneity of responses remain unclear.

Other studies in young populations illustrate the heterogeneity of responses between PM exposure and HRV. In a study of mail carriers between 25 and 46 years of age in Taiwan, no significant HRV effects from PM (mean sample time of approximately four hours using a personal cascade impactor sampler with a pump) were observed despite reported higher traffic-related air pollution exposure levels as compared to other studies (median PM_2.5_ exposure = 61.3 *μ*g/m^3^) [51]. Similarly, PM_2.5_ exposure assessed by a light-scattering method (pDR real time instrument, 0–180 min averaging time) did not affect the SDNN measurements in 40 young, healthy residents of the Mexican metropolitan area [52]. In a population of nine young highway patrol officers (mean age = 27.3 years), Riediker et al. [28] showed that in-vehicle exposure to PM_2.5_ (measured both by gravimetric and light-scattering methods) was associated with all time domain parameters (% difference between adjacent normal RR intervals that are greater than 50 msec or PNN50, SDNN, and mean cycle length, a phrase to emphasize that the interval between consecutive beats, rather than the heart rate, is being analyzed), as well as HF power and the power ratio LF/HF on the morning after the shift. In the occupational literature, Magari et al. [23] reported statistically significant associations using a two-hour lagged mean heart rate. Specifically, the PM_2.5_ average air concentration (measured using a light scattering instrument, mean 1160 μg/m^3^) showed an average increase of 1.67 msec (95% CI: 0.11 to 3.22) in the mean heart rate, for every 1 mg/m^3^ increase in the average PM_2.5_ concentration. The variety of exposure assessment techniques and corresponding averaging times may also contribute to the perceived heterogeneity of responses.

In addition to different PM exposure metrics, studies have used various methods to estimate the impact of gaseous co-pollutants. Some have used fixed-site monitors [13,20,30,43] and/or time activity data [43] to estimate traffic-related ambient air pollution exposure, including gaseous co-pollutants. Our results showing acute reductions in HRV in association with ozone and other gaseous pollutants is in agreement with other recent studies [30].

In a repeated measures study of 46 subjects (43–75 years of age), Zanobetti et al. [43] suggested that pollutant mixtures may influence cardiac tone as both PM and O_3_ (30–120 min moving averages) had independent associations with reduced HRV in two-pollutant models. Using a five minute time resolution to evaluate the acute effects of residential outdoor ozone exposure and HRV changes, Jia et al. [30] showed in 20 elderly subjects that, after adjusting for other pollutants and subject characteristics, there was a reduction in the HF component of 4.87% (95% CI:0.97 to 8.62) per 10 ppb increment of O_3_. A similar result was observed in a study of patients recently discharged from the hospital for acute coronary disease-related complications. Here, the two-hour and five-day O_3_ moving averages were associated with reductions in time domain components of HRV indicative of parasympathetic function, whereas NO_2_ was associated with reductions in the HF spectral component [53].

A major limitation of these studies (as referenced in both) was the use of fixed-site monitors to estimate personal exposure measurements. Suh and Zanobetti [54] showed that changes in HRV – especially those associated with parasympathetic control – were significantly and negatively associated with elemental carbon, and, to a lesser degree, NO_x_ when measurements of personal exposure (but not ambient, outdoor or indoor concentrations) were used to estimate their exposures. Interestingly and importantly, associations between personal exposure measurements and HRV were detectable only for these traffic-related pollutants. Non-significant findings with HRV were detected for 24-hour ambient concentrations and personal exposures to more spatially uniform regional pollutants, i.e. PM_2.5_ and O_3_.

Similar results were reported in a large controlled-exposure study examining the association between HRV and combined exposure to concentrated ambient particles (CAPs) and O_3_. In the participating group of healthy young adults (*n* = 50, mean age = 27.1 years), no consistent pattern of changes in HRV indices was detected among PM_2.5_ and O_3_ exposure categories [21]. Despite the absence of a clear trend in the categorical exposure models, the dose–response analysis demonstrated a trend toward a negative linear association between CAPs mass concentration and change in several HRV indices, with a statically significant relationship for the LF HRV measure. No relationship existed without accounting for O_3_.

Given the similarities between these studies with our results, it is possible to speculate that, regardless of age or underlying cardiovascular disease, acute ozone exposures are associated with reductions in HRV. With respect to acute PM_2.5_ exposures, there are notable age-dependent and/or possibly underlying cardiovascular diseases that may influence the HRV responses to PM. In addition, acute changes in HRV (even those characterized by an increase in the HF domain) can be associated with the onset of a ventricular arrhythmia [49].

To the best of our knowledge, the literature contains varied to little information on the relationship between HRV parameters and potential associations between NO_x,_ CO_2_, and formaldehyde. The selection of exposure measures reported here was not based on an a priori reason that some evidence existed to support association between exposure and HRV. Rather we evaluated potential associations within available exposures as part of the Mexico City Air Pollution Campaign. In 2004, the Multi-Ethnic Study of Atherosclerosis and Air Pollution was launched to investigate the relation between individual-level estimates of long-term air pollution exposure and the progression of subclinical atherosclerosis and the incidence of cardiovascular disease and includes exposure assessments of fine particulate matter, NO_x_, and black carbon; the majority of data collection will be completed in 2014 [55]. Some have shown that certain nitrogen oxides, e.g. NO_2_, do not affect heart rate variability at concentrations high for urban background levels and in the absence of other pollutants [56]. Others have shown that ambient NO_2_ concentrations were inversely associated with SDNN and positively associated with LF/HF. (β = 1.4; 95% CI, 0.35 to 2.5) 2 hr after the start of cycling [57]. Our results demonstrate that both NO_2_ and NO_x_ are significantly associated with SDNN. No studies were located that demonstrated a cardiovascular effects in humans after inhalation exposure to CO_2_ or formaldehyde [58]. In our case, it’s likely that CO_2_ and/or formaldehyde exposures were acting as a surrogate for other vehicular gaseous pollutants.

One limitation of this work is that this analysis did not control for stress resulting from traffic. Psychological stress can influence both HRV and autonomic function [59]. Since we did not measure participant stress while in traffic, we cannot disentangle the impact of both pollution and stress exposures resulting from being in traffic. In addition to traffic-related stress, participants may have observed the measured pollutant concentrations while being transported in the van-based mobile laboratory. Knowledge of their exposures may have stimulated the participants' sympathetic tone. Secondly, any exposure misclassification can lead to biased estimates of exposure-response estimates, particularly in cases with multiple correlated exposures where the direction of the bias is uncertain [60]. While exposure error biases are often assumed to bias toward the null, a more complicated situation arises in cases like ours when two or more exposures are measured with error and are correlated with each other. This may lead to bias in both directions and with varying degree. A final caveat is that chemical exposure factors are known to affect HRV [61,62], but these exposures are unlikely to be present in our study and thus, would be unlikely to affect our findings.

## Conclusions

This study revealed an association between traffic-related PM_2.5_ exposure and acute changes in HRV in a population aged 22 to 56 years when PM_2.5_ exposures were relatively low (14 *μ*g/m^3^). Results also demonstrate heterogeneity in the effects of the different pollutants, with declines in HRV – especially HF – with ozone and formaldehyde exposures, and increases in HRV with PM_2.5_ exposure. Our findings support the need for additional research emphasizing the impacts of co-pollutants [63]. Given that exposure to traffic-related emissions has been associated with increased risk of cardiovascular morbidity and mortality, understanding the mechanisms by which traffic-related emissions can cause cardiovascular disease has significant public health relevance.

## Abbreviations

CAPs: Concentrated ambient particles; CO2: Carbon dioxide; CO: Carbon monoxide; HF: Power in high frequency; HCHO: Formaldehyde; HRV: Heart rate variability; IQR: Interquartile range; LF: Low frequency; NAAQS: National Ambient Air Quality Standards; NDIR: Non-dispersive infrared; NO2: Nitrogen dioxide; NOx: Nitrogen oxides; O3: Ozone; PM2.5: Fine particulate matter; SDNN: Standard deviation of normal-to-normal intervals; TILDAS: Tunable infrared laser differential absorption spectrometer.

## Competing interests

The authors declare that they have no completing interests.

## Authors’ contributions

KNS drafted the manuscript. JC and MOH performed the statistical analysis. ML performed the field work. LM and MM provided logistical and financial support. FH conceived of the study and participated in its design and coordination. All authors read and approved the final manuscript.
